# A cognitive behavioural intervention for low self‐esteem in young people who have experienced stigma, prejudice, or discrimination: An uncontrolled acceptability and feasibility study

**DOI:** 10.1111/papt.12361

**Published:** 2021-08-29

**Authors:** Katie Langford, Katrina McMullen, Livia Bridge, Lovedeep Rai, Patrick Smith, Katharine A. Rimes

**Affiliations:** ^1^ Department of Psychology Institute of Psychology, Psychology & Neuroscience King’s College London UK; ^2^ South London and Maudsley NHS Foundation Trust, Bethlem Royal Hospital Beckenham UK

**Keywords:** cognitive behavioural therapy, compassion‐focussed therapy, discrimination, self‐esteem, stigma

## Abstract

**Objectives:**

Stigma has been found to be associated with lower self‐esteem, which increases the risk of difficulties across life domains including vulnerability to mental health problems. There are no previous studies of interventions for people experiencing low self‐esteem in the context of different stigmatized characteristics. This study evaluated feasibility, acceptability, and preliminary outcomes of an intervention targeting low self‐esteem in stigmatized people aged 16–24 years.

**Design:**

An uncontrolled study with repeated measures.

**Method:**

People with a range of stigmatized characteristics, who had low self‐esteem and associated impaired daily functioning, were recruited from the general population. The individual six‐session cognitive behavioural intervention had modules chosen according to participants’ formulations. The CBT included compassion‐focussed therapy methods and was informed by stigma research. Feasibility was assessed in relation to recruitment, retention, and protocol adherence. Acceptability was assessed through participant feedback. Questionnaires assessing self‐esteem, functioning impairments, depression, anxiety, self‐criticism, self‐compassion, and responses to prejudice and discrimination were administered at baseline, pre‐, mid‐, post‐intervention, and two‐month follow‐up.

**Results:**

Forty‐four people completed screening; 73% were eligible. Of these, 78% consented and 69% (*N* = 22) started the intervention. Eighteen (82%) participants completed, and four dropped out. Follow‐up measures were completed by all treatment completers. Treatment completers reported the intervention was useful, improved their self‐esteem and coping, and would recommend it. Ratings of usefulness and frequency of use of intervention components were high at post‐treatment and follow‐up.

**Conclusions:**

The intervention was feasible and highly acceptable to treatment completers. This suggests the intervention warrants investigation in a randomized‐controlled trial.

**Practitioner points:**

- Young people with low self‐esteem whom have been negatively affected by stigma may wish to access support and be willing to engage in psychological interventions.
- Cognitive behavioural therapy may be helpful for young people with low self‐esteem who have experienced stigma, prejudice, or discrimination.
- Cognitive behavioural techniques such as self‐compassionate thought records and behavioural experiments were considered acceptable and helpful by young people whose self‐esteem has been affected by stigma.
- Addressing responses to stigma in therapy, such as rumination, avoidance, and perfectionism, appears to be feasible and acceptable.

## Background

Stigma is the devaluation of a group or individual based on a characteristic that is discredited by society (Goffman, 2009), such as having a mental illness, physical health condition, minority sexual orientation or gender identity, female sex, higher body weight, disability, and minority ethnic/racial heritage. The intersectionality literature highlights how different characteristics interact and overlap to affect an individual’s experiences of discrimination and privilege (Crenshaw, 1989). Stigma processes include negative attitudes, opinions, and feelings (prejudice), unfair treatment when these attitudes are acted upon (discrimination), and internalization of negative stereotypes, known as self‐stigma (Corrigan & Watson, 2002). Theories of stigma have proposed that harm to self‐esteem is a possible consequence (Corrigan et al., 2009; Link, Cullen, Struening, Shrout, & Dohrenwend, 1989; Major & O’Brien, 2005). Low self‐esteem means a low opinion of oneself and lack of self‐worth. Lower self‐esteem has been observed in stigmatized groups, for example people with minority sexual orientation (Bridge, Smith, & Rimes, 2019), higher body weight (Sikorski et al., 2015), and psychosis (Bradshaw & Brekke, 1999). Cross‐sectional studies have found associations between self‐stigma and low self‐esteem (Austin & Goodman, 2017; Corrigan et al., 2006; Durso et al., 2012). Longitudinal studies have found evidence for a causal role for stigma processes in the reduction of self‐esteem (Greene, Way, & Pahl, 2006; Link, Struening, Neese‐Todd, Asmussen, & Phelan, 2001). Discrimination experiences in adolescence have been associated with lower self‐esteem in adulthood (Yang, Chen, Choi, & Kurtulus, 2019), suggesting that stigma‐related experiences in early life may have lasting effects on self‐esteem. Low self‐esteem is typically viewed as a transdiagnostic construct in relation to mental illness. Not only might it be a symptom, consequence, or maintaining factor for mental illness but also there is evidence that low self‐esteem can be a vulnerability factor for depression (Orth, Robins, & Widaman, 2012; Ju & Lee, 2018), psychosis (Krabbendam et al., 2002), eating disorders (Cervera et al., 2003), social anxiety (van Tuijl, de Jong, Sportel, de Hullu, & Nauta, 2014), and other psychosocial problems (Trzesniewski et al., 2006). Low self‐esteem is associated with negative outcomes in other life domains including relationship and job satisfaction (Orth, Robins, & Widaman, 2012).

Research suggests that responses and coping styles may increase vulnerability or buffer against effects of stigma (Miller & Kaiser, 2001). Group attachment and external attribution of blame can be protective for self‐esteem (Bourguignon, Seron, Yzerbyt, & Herman, 2006; Crocker & Major, 1989). Poorer outcomes have been associated with avoidance and withdrawal, compared to using social support and proactive coping (Aristegui, Radusky, Zalazar, Lucas, & Sued, 2018; McDermott, Umaña‐Taylor, & Zeiders, 2019). Rumination has been found to mediate the relationship between stigma‐related stress and psychological distress (Hatzenbuehler, Nolen‐Hoeksema, & Dovidio, 2009).

Interventions for low self‐esteem include cognitive behavioural therapy (CBT) based on Fennell’s (1997) model, which aims to address factors that maintain low self‐esteem, such as self‐critical thinking and avoidance, and underlying core beliefs and unhelpful attitudes (Fennell, 1998). A meta‐analysis of interventions based on Fennell’s model found strong effect sizes for improvements in self‐esteem and reductions in depressive symptoms (Kolubinski, Frings, Nikčević, Lawrence, & Spada, 2018). Other forms of cognitive behavioural interventions may also be helpful. Rose, McIntyre, and Rimes (2018) found significant improvements in self‐esteem following a six‐session compassion‐focussed intervention for highly self‐critical university students. As far as the authors are aware, there have been no investigations of cognitive behavioural (or other) interventions targeting low self‐esteem in people with a range of stigmatized characteristics.

### Aims

The current study evaluated a new cognitive behavioural intervention for low self‐esteem, tailored for people aged 16–24 years who had experienced stigma, prejudice, and/or discrimination. Young adults were targeted as a relatively early intervention approach with individuals who had sufficient life experience to be aware of the impact of stigma and low self‐esteem on their life.

The following research questions were explored:

1. Is the intervention feasible to deliver in terms of recruitment, retention, and protocol‐adherence?
2. Do participants find the study process and intervention acceptable?
3. Compared to pre‐intervention, at post‐intervention and follow‐up, do participants report higher self‐esteem, lower functional impairments related to self‐esteem, and reductions in depression and anxiety?
4. Do participants show improvements in measures of potential processes of change; self‐criticism, higher self‐compassion, and coping responses related to stigma? Are changes in self‐esteem associated with improvements in these process measures?

## Method

### Ethical statement

Ethical approval was obtained from the local university’s Research Ethics Subcommittee.

### Design

This was an uncontrolled study with repeated measures at baseline (at least 2 weeks prior to session 1), pre‐intervention (session 1), mid‐intervention (after session 3), post‐intervention (after session 6), and two‐month follow‐up. Qualitative and quantitative data were collected to investigate feasibility and acceptability of the intervention, study process, and assessment methods. Standardized self‐report measures were collected to provide preliminary information about pre‐ to post‐treatment changes. Randomization was not included because the purpose was to undertake primary data collection relating to feasibility and acceptability of the novel intervention, as the first stage of piloting within the process of intervention development, informed by MRC recommendations framework for development of complex interventions (O'Cathain et al., 2019).

### Participants

People aged 16–24 years, who identified that stigma, prejudice, or discrimination had negatively impacted their self‐esteem, were recruited from the general population. To participate they had to score under 25 on the Rosenberg Self‐Esteem Scale (Isomaa, Väänänen, Fröjd, Kaltiala‐Heino, & Marttunen, 2012; Rosenberg, 1965), report that low self‐esteem was causing significant impairment in daily functioning as indicated by a score of 10 or above on the Work and Social Adjustment Scale (Mundt, Marks, Shear, & Greist, 2002), and have sufficient proficiency in English. They were excluded if they were receiving another psychological intervention, had started or changed dose of psychoactive medication in the past three months, met criteria for a current serious mental health problem that would be more appropriately treated in NHS services (e.g. schizophrenia, bipolar disorder, or anorexia nervosa), reported suicidal ideation with intent or plan to act on suicidal thoughts, or reported recent self‐harm. The target sample size was 16–25 participants, in line with recommendations for pilot intervention studies (Hertzog, 2008).

### Procedure

Recruitment took place from April to November 2019, with two researchers working 1–2 days per week on the study; follow‐ups were completed in April 2020. The study was advertised primarily online, using social media, research recruitment registers, and circular emails inviting volunteers for university research projects. Potential participants accessed the information sheet online, registered their interest, and were provided with screening questions to complete online. If eligible based on screening questions, they were invited for telephone screening. If not eligible, participants were signposted to sources of support where appropriate. Following telephone screening, eligible participants completed the consent form and baseline questionnaires and were offered an appointment for session 1 at least two weeks later. Links to questionnaires were sent to participants from a study email account, and email reminders prompted participants if they were uncompleted.

### Intervention

Two trainee clinical psychologists delivered the intervention, supervised by two consultant clinical psychologists in weekly group supervision. Therapists were in their second and third years of training, had CBT experience, had learned about compassion‐focussed therapy (CFT), and were involved in intervention development. Six 1‐hour individual sessions were delivered weekly, with booklets to supplement learning (see Table 1 for intervention content). The intervention was informed by stigma theory and research. It was based on CBT principles including Fennell’s (1997) model of low self‐esteem and Gilbert’s Compassionate Mind approach (Gilbert, 2009). The CFT ‘threat/safety strategy’ formulation (Gilbert, 2010) was used to identify experiences contributing to key fears (known as ‘core beliefs’ in traditional CBT) about self and others, such as discrimination experiences, current coping strategies, and their intended and unintended consequences. Gilbert’s ‘three systems’ model of emotion regulation (Gilbert, 2009) was used throughout the intervention. These existing approaches were adapted to be tailored to people with stigmatized characteristics. For example, psychoeducation was provided about the activation of the ‘Threat’ system in response to stigma, and internalization of negative attitudes (self‐stigma) in development and maintenance of low self‐esteem. Each session incorporated stigma‐specific explanations and examples. The first two sessions were adapted from Rose et al. (2018) to address self‐criticism. Session 4 focussed on developing more helpful beliefs as alternatives to key fears about the self. Optional ‘modules’ for sessions 3 and 5 corresponded to unhelpful processes that were found to be associated with low self‐esteem reported by stigmatized individuals in previous research, for example rumination and avoidance. The modular approach had the advantage of tailoring sessions to participants’ formulations. Every session was audio‐recorded and listened to by the therapists’ supervisor, to assess fidelity and for supervision purposes.

**Table 1:** Core and optional modules for each session of the cognitive behavioural intervention for low self‐esteem in young people with stigmatized characteristics

Core modules	Description of module
Formulation of low self‐esteem in the context of stigma and identifying self‐critical thinking (Session 1)	Collaborative formulation of how low self‐esteem developed and is maintained in the context of stigmatized characteristics (e.g. highlight role of discrimination experiences and self‐stigma in development of negative beliefs about self, world, and others) Psychoeducation regarding self‐esteem, ‘three systems’ model of emotion regulation; how stigma activates the threat system; self‐compassionate approach (Gilbert, 2009) Introduction to self‐criticism and monitoring of self‐critical thinking for homework
Self‐compassion as an alternative to self‐criticism (Session 2)	Introduction of self‐compassion as an alternative to self‐criticism, including in response to experiences of stigma Completion of a self‐compassionate thought record in session and for homework Introduction of compassionate imagery exercise for homework
Addressing Key Fears (Session 4)	Psychoeducation about link between stigma, low self‐esteem, and overgeneralized negative beliefs about the self and/or others (core beliefs, called ‘key fears’ in this intervention) Updating key fear with new information Identifying more helpful belief as alternative to key fear Set up positive data log and behavioural experiment
Therapy summary/goal setting for follow‐up period (Session 6)	Review of previous sessions and techniques Therapy summary including overview of main learning points and planning for situations where self‐esteem might be more vulnerable or stigmatizing experiences may occur Goal setting for between Session 6 and two‐month follow‐up
Follow‐up telephone session (2 months after session 6)	Review current level of self‐esteem, goals/plan and each intervention strategy Option to discuss one strategy in more detail Relapse prevention plan Signposting to further support if necessary
**Optional modules (Sessions 3 & 5)**	**Description of module**
Dealing with avoidance^a^	Collaborative discussion about role of avoidance in maintenance of low self‐esteem Psychoeducation about avoidance, including as a response to stigma, prejudice, or discrimination Identify alternatives (approach behaviours); set up behavioural experiment/graded exposure
Reducing overthinking^a^	Collaborative discussion about role of overthinking in maintenance of low self‐esteem Psychoeducation about overthinking (worry/rumination), including in response to stigma, prejudice, or discrimination Identifying alternatives (distraction, present moment focus, move towards action, e.g. group connection/activism) and set up practice for homework Problem solving (optional for participant)
Letting go of very high standards	Identify perfectionist standards as coping strategy for having a stigmatized characteristic and how this maintains low self‐esteem Identify pros/cons of perfectionist standards and behaviours Explore current perfectionist standard and identify alternative Set up behavioural experiment If time: plan to expand another valued area of life
Social comparisons, social media use, and role models^a^	Collaborative discussion about social comparisons and social media use, including in relation to stigma, and how these factors can maintain of low self‐esteem Psychoeducation about consequences of social comparison Identify positive social media use (e.g. connecting with others who share stigma experiences; engaging with organizations that empower marginalized voices) Make plan for more helpful social media use Finding role models (optional)
Assertiveness^a^	Collaborative discussion about low assertiveness, including in the face of stigma, and how this contributes to maintenance of low self‐esteem Psychoeducation regarding communication styles Self‐compassionate thought record in relation to assertiveness (optional) Set up behavioural experiment to be assertive Role play assertive communication (optional to client)
Coping with unpleasant feelings^a^	Collaborative discussion about current distress tolerance strategies in maintenance of low self‐esteem Psychoeducation about distress tolerance and impact of avoiding emotions Strategies to accept/tolerate unpleasant feelings Set up behavioural experiment to try new strategy
Hiding part of ourselves^b^	Collaborative discussion about concealment of stigmatized characteristics in maintenance of low self‐esteem Psychoeducation about hiding stigmatized characteristics Cost/benefit analysis Hierarchy for safe disclosure or behaviour change Set up behavioural experiment to test feared disclosure or behaviour change
Building a support network^a^, ^b^	Collaborative discussion about reduced social support in the context of stigma, and maintenance of low self‐esteem Psychoeducation regarding purpose of support network Identification of barriers to accessing support Identification of at least one new source of support and set up plan for homework
Working with early memories^b^, ^c^	Identify negative/distressing memories linked to key fear and provide rationale for memory‐focussed techniques Discrimination training: identify differences between ‘then’ and ‘now’; practice for homework Imagery re‐scripting: ‘re‐live’ the event from participant’s perspective and insert new meaning or information (using updates completed in key fears module)

Note

^a^ Module can be shortened and combined with another module in sessions 3 and 5

^b^ Modules were prepared and offered but not delivered

^c^ Only to be delivered at Session 5 if negative images and memories from the past are problematic and linked to key fear.

### Feasibility and acceptability objectives

Feasibility was assessed by examining rates of recruitment, suitability of eligibility criteria, retention, and protocol adherence. Acceptability was assessed through participant feedback.

### Fidelity

Treatment fidelity was assessed by therapists’ primary supervisor. Session recordings were rated a dichotomous yes/no for whether therapists adhered to session protocol. Protocol violations were recorded and fed back to therapists.

### Measures

Standardized questionnaires were completed online. Clinical measures were administered at screening. Clinical and process measures were completed at baseline, pre‐intervention, mid‐intervention, post‐intervention, and two‐month follow‐up.

#### Screening tool for mental health problems

##### Mini International Neuropsychiatric Interview (MINI, version 7.0.2)

The MINI, a brief structured interview for major disorders in DSM‐5 and ICD 10, was administered at telephone screening to assess current mental health diagnoses (Sheehan et al., 1998).

#### Clinical measures

##### Rosenberg Self‐Esteem Scale (RSES)

The RSES has ten items assessing global self‐esteem; responses are on a 4‐point scale from 1 (‘strongly disagree’) to 4 (‘strongly agree’; Rosenberg, 1965). Five items provide a negative statement and are reverse scored. Total scores range from 10 to 40; higher scores reflect higher self‐esteem. Cronbach’s alphas were .55 to .91 (<70 at baseline and pre‐intervention).

##### Work and Social Adjustment Scale (WSAS)

The WSAS assesses day‐to‐day functioning in five domains: work, home management, social leisure activities, private leisure activities, and relationships (Mundt et al., 2002). Usual wording of the scale’s instructions was adapted (‘your problems’ replaced with ‘low self‐esteem’) to assess impact of low self‐esteem. Responses are on an 8‐point scale from 0 (‘not at all’) to 8 (‘very severely’). Total scores range from 0 to 40; greater scores indicate poorer functioning. Cronbach’s alphas were .69 to .89.

##### Patient Health Questionnaire (PHQ‐9)

The PHQ‐9 has nine items measuring depressive symptoms (Kroenke, Spitzer, & Williams, 2001). Response options range from 0 (‘not at all’) to 3 (‘nearly every day’). Higher scores indicate greater severity of depressive symptoms. Cronbach’s alphas were .72 to .90.

##### Generalized Anxiety Disorder (GAD‐7)

The GAD‐7 has seven items measuring symptoms of anxiety, on a scale from 0 (‘not at all’) to 3 (‘nearly every day’; Spitzer, Kroenke, Williams, & Löwe, 2006). Higher scores indicate greater severity of anxiety‐related symptoms. Cronbach’s alphas were .82 to .90.

#### Process measures

##### Forms of Criticism/Self‐Attacking and Self‐Reassuring Scale (FSCRS)

The FSCRS is a 22‐item instrument with a three‐factor structure (Castilho, Pinto‐Gouveia, & Duarte, 2013; Gilbert, Clarke, Hempel, Miles, & Irons, 2004). The ‘Inadequate Self’ subscale (nine items) measures self‐criticism related to perceived inadequacy and disappointment with oneself; ‘Hated Self’ (five items) measures self‐criticism relating to self‐hatred and self‐punishment; ‘Reassured Self’ (eight items) measures the ability to reassure and sooth oneself. Responses range from 0 (‘not at all like me’) to 4 (‘extremely like me’). Cronbach’s alphas: FSCRS‐IS.56 (baseline) −.96; FSCRS‐HS.31 (pre‐intervention) −.91; FSCRS‐RS .68 to .96.

##### Self‐Compassion Scale (SCS)

The SCS has 26 items; responses range from 1 (‘almost never’) to 5 (‘almost always’; Neff, 2003). A total SCS score can be used as an overall measure of self‐compassion (Neff, Whittaker, & Karl, 2017). Higher scores indicate greater self‐compassion. Cronbach’s alphas ranged from .88 to .96.

##### Discrimination and Prejudice Responses Scale (DAPR)

The DAPR assesses coping responses associated with prejudice and discrimination, with 11 components representing different categories of responses, each with four items (Armstrong, Henderson, & Rimes, 2019). Subscales are Preparation (e.g. ‘ready myself for encountering prejudice or discrimination’), Raise Awareness (‘educate people about the characteristic(s) to increase their understanding’), Avoidance (‘avoid people who I know to be prejudiced’), Enjoyable Activity (‘do an activity that makes me feel good’), Group Attachment (‘identify more closely with other people with the characteristic(s)’), Secrecy (‘hide the characteristic(s) from people’), Self‐reliance (‘rely on myself more than others’), Distancing (‘change my behaviour to avoid being stereotyped by people’), Rumination (‘go over and over what I could have done differently during the event’), Resignation (‘put up with the way I am treated’), and Blame (‘blame the individual(s) involved for their behaviour rather than myself’). Responses range from 1 (‘never’) to 5 (‘always’). Cronbach’s alphas were Resignation: .32 to .72 (<70 at all time points except baseline); Avoidance: .60 (mid‐intervention) −.81; Distancing: .68 to .92; Rumination: .70 to .97; Blame: .72 to .92; Raise Awareness: .81 to .95; Enjoyable Activity: .83 to .96; Group Attachment: .87 to .92; Self‐reliance: .78 to .86; Preparation: .85 to .97; Secrecy: .90 to .98.

#### Feedback

Participant feedback was collected at post‐intervention and follow‐up, with quantitative rating scales and open‐ended questions, adapted from Rose et al. (2018). Post‐intervention feedback included general questions about the intervention and study process, and ratings of usefulness of intervention elements. Post‐intervention, participants reported the average proportion of weekly booklets they had read, and average time spent on weekly homework. At follow‐up, ratings of usefulness and frequency of use of elements of the intervention were collected.

Qualitative feedback was collected from therapists and supervisors after intervention delivery was completed.

### Data analyses

Descriptive statistics were conducted to examine feasibility and quantitative feedback items. Open‐ended feedback was analysed using brief content analysis (Mayring, 2019).

Descriptive statistics and within‐participants effects (effect sizes and 95% confidence intervals) were conducted to examine changes in scores for each measure. Changes are presented for the eighteen treatment completers. Change scores were calculated for the following time points: baseline to pre‐intervention; pre‐intervention to post‐intervention; pre‐intervention to follow‐up; post‐intervention to follow‐up. Effect sizes were calculated using Cohen’s *d_z_* for repeated measures and interpreted using the following cut‐offs: ‘negligible’ effect <0.2; small effect ≥0.2, medium effect ≥0.5, large effect ≥0.8.

Reliable Change Index (RCI) was used to discover whether participants made reliable improvements and reliable recovery (Jacobson & Truax, 1991). The proportion of participants who made reliable improvements was calculated, and the proportion of participants who made reliable recovery was calculated for RSES and WSAS. Reliable recovery was not examined for PHQ‐9 and GAD‐7 because participants did not all begin the intervention in the clinical range.

Associations between pre‐ to post‐intervention change scores and clinical and process measures were explored using Spearman’s correlations.

## Results

### Participant characteristics

Twenty‐two participants began the intervention. Their characteristics are shown in Table 2. Participants’ mean age was 21.9 years. Participants were mostly female (*n* = 20, 90.9%) and fulltime students (*n* = 16, 72.7%). Participants were ethnically diverse; the largest ethnic group was White (*n* = 7, 31.8%). Participants reported a range of stigmatized characteristics; most common were race/ethnicity and having experienced a mental health condition. Three participants (13.6%) reported one characteristic, most reported two or three (total *n* = 18, 81.8%). One participant reported four characteristics (4.5%). Most participants met criteria for at least one psychiatric disorder at screening and had received psychological therapy before.

**Table 2:** Characteristics of participants who started the intervention (*n* = 22)

Characteristics	
Age, mean (*SD*, range), years	21.3 (1.9, 18–24)
Sex, *n* (%)	
Female	20 (90.9)
Male	2 (9.1)
Ethnicity, *n* (%)	
Asian	4 (18.2)
Black	3 (13.6)
Mixed/multiple ethnic groups	5 (22.7)
Other ethnic group	3 (13.6)
White	7 (31.8)
Highest education achievement	
A‐levels	1 (4.5)
Undergraduate/Bachelors (current)	14 (63.6)
Undergraduate/Bachelors (obtained)	5 (22.7)
Postgraduate/Masters (current)	2 (9.1)
Self‐identified stigmatized characteristics, *n* (%)	
Race/ethnicity	8 (36.4)
Mental health condition	8 (36.4)
Body weight	7 (31.8)
Other physical appearance	7 (31.8)
Sexual orientation	5 (22.7)
Religion	4 (18.2)
Sex (female)	5 (22.7)
Physical health condition	3 (13.6)
Class/socioeconomic status	2 (9.1)
Learning difficulty (dyslexia or dyspraxia)	2 (9.1)
Gender identity	1 (4.5)
Psychiatric diagnoses, *n* (%)	
None	3 (13.6)
Major depressive episode	
Current	14 (63.6)
Past	2 (9.1)
Panic disorder	
Current	2 (9.1)
Lifetime	2 (9.1)
Social anxiety disorder (Current)	7 (31.8)
Alcohol use disorder (Past)	1 (4.5)
Psychotic disorder (Lifetime)	1 (4.5)
Bulimia nervosa	
Current	4 (18.1)
Past	1 (4.5)
Binge‐eating disorder (Current)	1 (4.5)
Generalized anxiety disorder (Current)	6 (27.3)
Past psychological therapy, *n* (%)	
Cognitive behavioural therapy	5 (22.7)
Counselling	4 (18.2)
Group dialectical behavioural therapy	1 (4.5)
Group therapy, unspecified type	1 (4.5)
Other individual psychotherapy	3 (13.6)
Past psychological therapy (any)	14 (63.6)

### Feasibility

**Figure 1:**
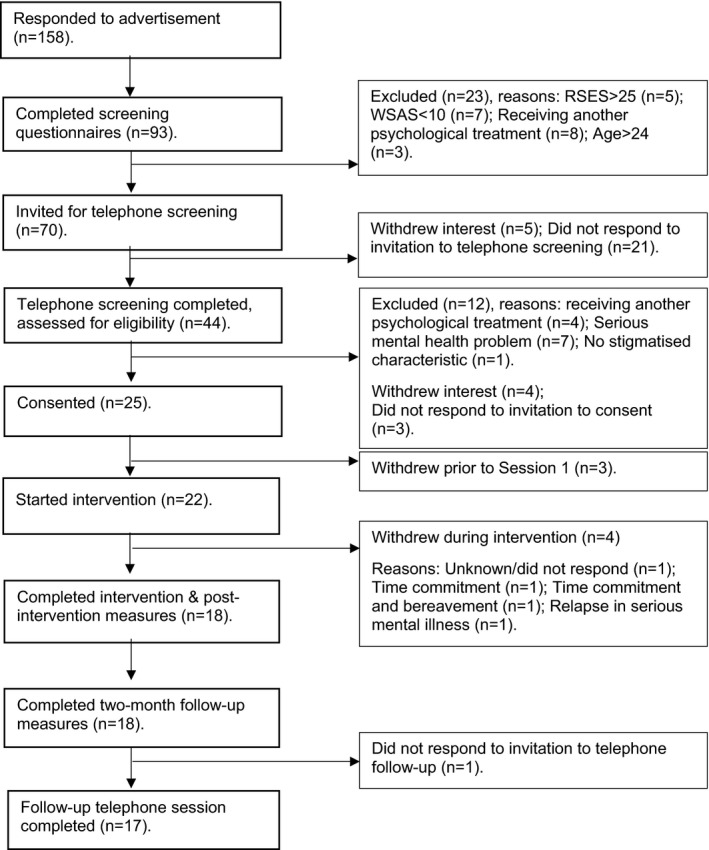
Flow diagram showing recruitment process and participant retention. RSES = Rosenberg Self‐Esteem Scale; WSAS = Work and Social Adjustment Scale.

Figure 1 summarizes details of recruitment, retention, and reasons for drop‐out. One hundred and fifty‐eight people responded to advertisements, 93 completed online screening questions, and 44 completed telephone screening. Of the 25 people who consented, 22 began the intervention. Four participants dropped out during the intervention. Eighteen completed all 6 sessions and measures. Eligibility criteria mostly identified suitable participants. One participant withdrew after session 5 due to a relapse in a serious mental health condition. One person dropped out without giving a reason, a participant with autism spectrum disorder (ASD).

The mean duration of intervention was 7.6 weeks (*SD* = 1.6). Mean percentage of weekly booklets read was 67.9% (*SD* = 27.4), and mean time spent on weekly homework was 63.7 min (*SD* = 36.5). Questionnaires were completed fully at each time point by all treatment completers.

### Acceptability

The feedback questionnaire was completed by 18 treatment completers and one participant who dropped out after session 3. In response to general questions about the intervention with forced‐choice responses, 100% ‘agreed’ or ‘strongly agreed’ that the intervention was useful and that they would recommend it to others (*n* = 19). Most participants ‘agreed’ or ‘strongly agreed’ that the intervention improved their self‐esteem (*n* = 16, 84.2%), that it improved their ability to cope with their self‐esteem (*n* = 18, 94.7%), and that their facilitator understood their needs and difficulties (*n* = 18, 94.7%).

Optional modules most frequently delivered were ‘Overthinking’ (*n* = 12), ‘Letting go of very high standards’ (*n* = 10), and ‘Avoidance’ (*n* = 10). Less common were ‘Social comparisons, social media use, and role models’ (*n* = 4), ‘Assertiveness’ and ‘Coping with unpleasant feelings’ (both *n* = 2). Modules available but not delivered, being considered less important in the maintenance of these participants’ low self‐esteem and associated impairments, were ‘Hiding a stigmatized characteristic’, ‘Building a support network’, and ‘Working with early memories’. Usefulness results are presented in Table 3. Most participants provided high post‐treatment usefulness ratings (‘quite a lot’ or ‘very much’) for monitoring self‐criticism (*n* = 17, 89.5%), compassionate thought records, compassionate behaviours, and addressing key fears (all *n* = 15, 78.9%). Post‐treatment usefulness ratings of optional modules were on average ‘quite a lot’ or ‘very much’ (medians all 3.0–4.0). Usefulness ratings remained moderate or high at follow‐up.

**Table 3:** Usefulness ratings of each element of the intervention at post‐intervention and follow‐up; post‐intervention data from all completers and one drop‐out (*n* = 19); follow‐up data from participants who completed follow‐up session (*n* = 17)

Technique or module	Completed module (*n*)	Time point	0 – Not at all, *n* (%)	1 – A little, *n* (%)	2 – Somewhat, *n* (%)	3 – Quite a lot, *n* (%)	4 – Very much, *n* (%)	N/A, *n* (%)	Median
Core modules/techniques									
Compassionate reframe/thought record	19	Post‐intervention	0	0	3 (15.8)	5 (26.3)	10 (52.6)	1 (5.3)	4.0
		Follow‐up	0	0	1 (5.9)	8 (47.1)	7 (41.2)	1 (5.9)	3.0
Compassionate behaviours	19	Post‐intervention	0	0	4 (21.1)	6 (31.6)	9 (47.4)	0	3.0
		Follow‐up	0	0	2 (11.8)	11 (64.7)	4 (23.5)	0	3.0
Self‐criticism monitoring^a^	19	Post‐intervention	0	0	2 (10.5)	7 (36.8)	10 (52.6)	0	4.0
Addressing key fears module	19	Post‐intervention	0	0	3 (15.8)	3 (15.8)	12 (63.2)	1 (5.3)	4.0
		Follow‐up	0	0	1 (5.9)	6 (35.3)	9 (52.9)	1 (5.9)	4.0
‘Compassionate Other’ imagery	19	Post‐intervention	3 (15.8)	4 (21.1)	5 (26.3)	1 (5.3)	3 (15.8)	3 (15.8)	2.0
		Follow‐up	0	1 (5.9)	0	1 (5.9)	2 (11.8)	13 (76.5)	3.5
Optional modules									
Overthinking	12	Post‐intervention	0	0	0	5 (26.3)	7 (36.8)	7 (36.8)	4.0
		Follow‐up	0	0	4 (23.1)	3 (17.6)	5 (29.4)	5 (29.4)	3.0
Letting go of very high standards	12	Post‐intervention	0	0	2 (10.5)	2 (10.5)	6 (31.6)	9 (47.4)	4.0
		Follow‐up	0	2 (11.8)	1 (5.9)	4 (23.1)	3 (17.6)	7 (41.2)	3.0
Avoidance	10	Post‐intervention	0	1 (5.3)	1 (5.3)	0	8 (42.1)	9 (47.4)	4.0
		Follow‐up	0	0	2 (11.8)	4 (23.1)	3 (17.6)	8 (47.1)	3.0
Social comparison, social media, role models	4	Post‐intervention	0	0	1 (5.3)	1 (5.3)	2 (10.5)	15 (78.9)	3.5
		Follow‐up	0	0	0	3 (17.6)	1 (5.9)	13 (76.5)	3.0
Assertiveness	2	Post‐intervention	0	0	1 (5.3)	0	1 (5.3)	17 (89.5)	3.0
		Follow‐up	0	0	0	1 (5.9)	1 (5.9)	15 (88.2)	3.5
Coping with unpleasant feelings	2	Post‐intervention	0	0	0	1 (5.3)	1 (5.3)	17 (89.5)	3.5
		Follow‐up	0	0	0	0	2 (11.8)	15 (88.2)	4.0

Note

^a^ Only asked at post‐intervention.

Frequency ratings at follow‐up for use of techniques/modules were high; most participants reported using them at least weekly, except for ‘compassionate other’ imagery, which was only practised by four participants.

Open‐ended feedback was largely positive. The most common theme was greater awareness and control over unhelpful self‐criticism and developing greater self‐compassion. Approximately half of participants reported that they would have preferred additional sessions. Several commented that a choice of locations would have made sessions more convenient.

Feedback from therapists about their experiences delivering the intervention was positive, particularly for the modular structure enabling sessions to be delivered flexibly. The supervisors fed back that it would be beneficial for training cases to be completed prior to the intervention being delivered within the context of a trial.

### Fidelity

Of 120 sessions delivered in total, 94% were rated as adherent to treatment protocol.

### Changes in measures

Results of analyses investigating changes in standardized questionnaire scores are presented in Table 4 and summarized below.

**Table 4:** Clinical measures: *M* (*SD*), change scores, effect sizes (*d_z_*), and 95% confidence intervals (CI) for intervention completers (*n* = 18)

Measure	Base‐line	Pre	Mid	Post	Follow‐up	Pre‐intervention changes (baseline‐pre)			Post‐intervention changes (pre‐post)			Follow‐up changes (pre‐follow‐up)			Follow‐up only changes (post‐follow‐up)		
	*M* (*SD*)	*M* (*SD*)	*M* (*SD*)	*M* (*SD*)	*M* (*SD*)	Change score, *M* (*SD*)	ES (*d_z_*_)_	CI	Change score, *M* (*SD*)	ES (*d_z_*_)_	CI	Change score, *M* (*SD*)	ES (*d_z_*)	CI	Change score, *M* (*SD*)	ES (*d_z_*)	CI
RSES	19.6 (2.7)	20.1 (2.3)	24.1 (3.2)	27.5 (4.3)	28.0 (3.3)	0.5 (2.4)	0.2	−0.3 to 0.7	7.4 (4.0)	1.8	1.0–2.6	7.9 (3.2)	2.5	1.5–3.5	0.5 (3.4)	0.1	−0.3 to 0.5
WSAS	22.6 (7.6)	19.2 (6.2)	16.8 (6.8)	12.1 (7.0)	11.0 (7.3)	−3.4 (7.8)	0.4	−0.2 to 1.0	−7.1 (7.1)	1.0	0.4–1.6	−8.2 (6.8)	1.2	0.6–1.8	−1.1 (6.5)	0.2	−0.2 to 0.6
PHQ‐9	13.8 (6.4)	12.3 (5.7)	9.9 (6.2)	8.3 (4.9)	7.9 (4.2)	−1.5 (2.7)	0.6	0.3–0.9	−4.1 (4.9)	0.8	0.3–1.3	−4.4 (5.0)	0.9	0.4–1.5	−0.3 (3.8)	0.1	−0.3 to 0.5
GAD‐7	11.7 (4.7)	10.4 (4.7)	8.8 (4.5)	6.2 (3.8)	6.7 (4.0)	−1.3 (3.5)	0.4	0.0–0.8	−4.2 (4.3)	1.0	0.4–1.6	−3.7 (5.7)	0.6	−0.1 to 1.3	0.5 (4.0)	0.1	−0.4 to 0.6
FSCRS‐IS	29.8 (3.9)	30.6 (4.3)	24.1 (6.0)	17.1 (8.8)	17.8 (7.7)	0.7 (2.9)	0.3	−0.0 to 0.6	−13.5 (8.7)	1.6	0.8–2.4	−12.8 (6.9)	1.7	0.9–2.5	0.7 (7.2)	0.1	−0.3 to 0.5
FSCRS‐HS	11.2 (4.0)	10.0 (3.0)	6.6 (4.2)	5.2 (4.3)	5.2 (5.1)	−1.2 (3.4)	0.4	−0.1 to 0.9	−4.8 (5.0)	1.0	0.3–1.7	−4.8 (5.3)	0.9	0.2–1.6	0.1 (3.8)	0.1	−0.3 to 0.5
FSCRS‐RS	11.3 (3.8)	11.1 (4.8)	15.1 (5.3)	18.8 (8.0)	19.2 (6.1)	−0.3 (3.5)	0.1	−0.3 to 0.5	7.7 (8.3)	0.9	0.2–1.6	8.1 (6.9)	1.2	0.5–1.9	0.4 (5.1)	0.1	−0.2 to 0.4
SCS	51.7 (12.9)	52.8 (11.1)	69.3 (14.7)	81.5 (22.2)	82.3 (20.1)	1.1 (6.4)	0.2	−0.1 to 0.5	28.7 (23.5)	1.2	0.5–1.9	29.6 (19.4)	1.5	0.8–2.3	0.8 (15.0)	0.1	−0.2 to 0.4

Note

CI = 95% confidence interval; ES = Effect size; FSCRS‐IS/‐HS/‐RS = Forms of Self‐Criticizing/Attacking & Self‐Reassuring Scale – Inadequate Self/Hated Self/Reassured Self subscales; GAD‐7 = Generalized Anxiety Disorder; PHQ‐9 = Patient Health Questionnaire; RSES = Rosenberg Self‐Esteem Scale; SCS = Self‐Compassion Scale; WSAS = Work and Social Adjustment Scale.

#### Clinical measures

Self‐esteem increased during the pre‐intervention period with a small effect size and increased from pre‐ to post‐intervention and pre‐intervention to follow‐up with large effect sizes (*d_z_* = 1.8–2.5). Self‐esteem showed small improvements from post‐treatment to follow‐up. There were improvements in functional impairment, depression, and anxiety symptoms from pre‐ to post‐intervention and pre‐intervention to follow‐up.

Reliable improvements in self‐esteem were made by 16 (89%) participants post‐intervention and 15 (83%) at follow‐up. Reliable recovery was found for 14 participants (78%) at post‐intervention and 15 (83%) at follow‐up. For functional impairment, ten participants (56%) made reliable improvements at post‐intervention and nine (50%) at follow‐up. Reliable recovery was found for five (28%) at post‐intervention, and six (33%) at follow‐up. Reliable improvements in depression were made by seven (39%) post‐intervention and nine (50%) at follow‐up. For anxiety, reliable improvement was found for ten (55%) post‐intervention and nine (50%) at follow‐up.

### Process measures

There were large decreases in FSCRS–Inadequate Self and Hated Self, and increases in FSCRS‐Reassured‐Self and self‐compassion scores, from pre‐ to post‐intervention and pre‐intervention to follow‐up.

Discrimination and Prejudice Responses (DAPR) subscales that showed the largest changes from pre‐ to post‐intervention and pre‐intervention to follow‐up were reductions in Rumination, Resignation, Distancing, and Secrecy and increases in Enjoyable Activity, Blame, and Raise Awareness.

Pre‐ to post‐intervention changes in all FSCRS subscales and self‐compassion had strong correlations with changes in self‐esteem (*r*s = −.60 to −.81). For DAPR, reduced Rumination and increased Blame correlated moderately with changes in self‐esteem (*r*s = −.51 to .58). Changes in other subscales were not significantly correlated with changes in self‐esteem. Associations between changes in self‐esteem and all other standardized questionnaire measures are presented in supplementary tables (Tables S1 and S2).

## Discussion

This is the first study to assess acceptability and feasibility of a cognitive behavioural intervention targeting low self‐esteem in young people with stigmatized characteristics.

### Feasibility

An adequate number of participants were recruited, indicating that some young adults with stigmatized characteristics in the general population are sufficiently motivated by a desire to improve their self‐esteem to respond to advertisements. The recruitment target was reached in an eight‐month period with researchers working part‐time. Most participants met criteria for a psychiatric diagnosis at screening, suggesting that there is a considerable level of clinical need in some young people with low self‐esteem who experience stigma, prejudice, or discrimination, who are not accessing services. These findings indicate that if low self‐esteem is to be targeted as a form of early intervention for mental health problems, research with adolescents should be considered.

While the recruitment process was effective, there was significant drop‐out during screening, with 28% of people who registered interest completing both screening stages. One factor was the effort required by telephone screening, compared to the relative ease of online. While social media promotions reached a large pool of potential participants, advertisement through local universities was more effective for accessing people who were retained. A larger study could prioritize local and community‐based recruitment sources.

Most participants were female, consistent with evidence of lower levels of self‐esteem in females than males (Bleidorn et al., 2016) and a higher female prevalence of common mental health problems (Kuehner, 2016; Li & Graham, 2016). High female representation may reflect prevalence of sex‐based discrimination; five participants reported that sexism contributed to their low self‐esteem. Although the study was open to participants aged 16 upwards, and attempts were made to access 16–17‐year‐olds by recruiting through social media used by younger people (e.g. Instagram), all participants were aged 18–24 years and most were university students. Alternative recruitment methods may be necessary to reach younger people, non‐students, and men.

A range of stigmatized characteristics were reported by participants, and most identified multiple characteristics. This highlights the value of an intervention for people with a range of characteristics, which uses individualized formulation to understand their possible cumulative effect, and interaction between dimensions of disadvantage and privilege on an individual level, in line with the concept of intersectionality.

The inclusion criteria identified suitable participants. The intervention may require some adaptation for people with an ASD diagnosis or past psychosis; two participants with these characteristics withdrew. Further investigation is recommended before a larger study, including further pilot work and service‐user involvement.

The retention rate was high, with over 80% completing the intervention. High levels of protocol fidelity indicate that it can be delivered adequately by therapists with training in CBT and the techniques used in the intervention, combined with regular supervision.

Online collection of measures was feasible, with completion rates of 100% for all measures by treatment completers, which supports this procedure in a future study. This suggests that participants in this age group have access to digital devices and are willing to complete measures online, which supports the use of digital methods for data collection, particularly important in the context of the Covid‐19 pandemic.

Low internal reliability demonstrated for RSES, FSCRS‐IS, FSCRS‐HS, and DAPR Resignation subscale, calls into question the suitability of these measures. Low Cronbach’s alphas may result from the small sample size. RSES is a widely used measure of self‐esteem, and other DAPR subscales demonstrated adequate internal reliability, which supports the inclusion of these measures. In contrast, the FSCRS scales may be less useful for measuring self‐criticism. The Inadequate Self scale assesses a more general sense of inadequacy and includes items about emotions and thought processes (Gilbert et al., 2004). A future study could consider a more specific, single measure of self‐criticism, such as the Self‐Critical Rumination Scale (Smart, Peters, & Baer, 2016), which has been recommended in a systematic review of self‐criticism measures (Rose & Rimes, 2018).

### Acceptability

Participants’ feedback indicated that the intervention and study procedures were acceptable. Open‐ended feedback corresponded with high usefulness ratings of intervention elements and suggested that developing self‐compassionate thinking as an alternative to self‐criticism was an important process in the intervention. This indicates good acceptability for the use of CBT methods and CFT techniques utilized.

Optional modules most frequently identified as important for inclusion were ‘Overthinking’, ‘Letting go of very high standards’, and ‘Avoidance’, suggesting that these are unhelpful processes for young people with low self‐esteem who experience stigma. This is consistent with Fennell’s model of low self‐esteem (1997), which proposes self‐critical rumination, avoidance, and unhelpful compensatory strategies, such as perfectionism, as maintaining factors. Avoidance and overthinking have been highlighted as problematic ways of coping with stigma (Hatzenbuehler et al., 2009; Link et al., 1991). Perfectionism has also been considered an unhelpful coping strategy (Pachankis & Hatzenbuehler, 2013), although less frequently highlighted in the stigma literature than other processes. Modules that were prepared but not delivered (e.g. ‘Hiding a stigmatized characteristic’) were less relevant to participants’ formulations in this sample but may be beneficial in a future delivery of the intervention.

Several participants who indicated a preference for more sessions had additional mental health issues; offering additional sessions could be considered for participants with a higher level of clinical need or comorbid mental health conditions. Some participants would have liked a choice of locations, which may not be feasible, but future studies could consider online or video‐call‐based delivery to improve accessibility.

### Changes in self‐esteem and other measures

#### Clinical measures

Large effect sizes for improvements in self‐esteem, and high proportion of participants who made reliable improvements and recovery in self‐esteem, provide preliminary evidence that the intervention is worthy of further investigation. The effect sizes for self‐esteem are consistent with the large summary effect size reported in a meta‐analysis of weekly CBT for low self‐esteem (Kolubinski et al., 2018).

#### Process measures

The primary function of process measures was to evaluate their feasibility and suitability for assessing relevant processes in the intervention. For all the scales, changes were in the expected directions and there was evidence to suggest that they were sufficiently sensitive to change. The DAPR, which assesses responses to discrimination and prejudice, is a relatively new measure which has not been used in previous intervention research so this study provides promising results. A larger study could investigate whether changes in these process measures precede and mediate changes in self‐esteem.

However, changes found on the SCS should be interpreted with caution; the measure has been criticized for including three subscales assessing uncompassionate responses towards the self. The total score may reflect psychopathology rather than self‐compassion (Muris et al., 2018). Other self‐compassion measures should be reviewed for possible use in future research. As noted above, it is also suggested that alternative measures of self‐criticism are considered.

Improvements in self‐criticism and self‐compassion were consistent with findings of Rose et al. (2018) in their six‐session self‐criticism intervention. This, along with acceptability findings outlined above, supports the inclusion of compassion‐focussed interventions for stigmatized individuals with low self‐esteem. Moderate to strong correlations between improvements in self‐esteem and improvements in self‐criticism and self‐compassion suggest these may be potential mechanisms of change in the intervention.

Reductions in rumination were strongly correlated with improvements in self‐esteem. Previous studies have found that rumination is highly related to low self‐esteem (Kuster et al., 2012), and self‐critical thinking (O’Connor & Noyce, 2008). These findings and those for the DAPR are encouraging because coping responses have been found to be possible mediators of the potentially negative consequences of stigma (Major & O’Brien, 2005; Miller & Kaiser, 2001). However, it is unclear whether reducing these unhelpful processes contributed to improvements in self‐esteem, or whether as self‐esteem improved, participants felt able to use more helpful strategies. This requires clarification in a larger study.

### Limitations

Changes in measures must be interpreted with caution due to the uncontrolled study design; it is not possible to conclude that the observed changes were associated with the intervention. The study was not designed to assess efficacy and changes presented here only involved 18 treatment completers. Similarly, the acceptability outcomes are biased towards those participants who completed the intervention and were likely to have found it more helpful. Therapists had a dual role, delivering the intervention and collecting data, which may be another source of bias.

Participant characteristics of mainly female undergraduate students cannot necessarily be generalized to other young people, such as males and people who have received less education. There were only two therapists, who were involved in intervention design and had weekly supervision. Feasibility of therapists new to the intervention being able to deliver it adequately, in real‐world circumstances or NHS settings, including less intense supervision, requires investigation.

‘Lockdown’ during the COVID‐19 pandemic may have impacted on five participants’ outcomes at follow‐up. At follow‐up, several participants reported increased anxiety and low mood, and social distancing measures were preventing them from continuing to work on their self‐esteem, for example limiting opportunities to socialize. This context should be considered when interpreting follow‐up findings.

### Future research

The current study provides the basis for a future trial of the intervention. It is recommended that the next stage of investigation includes randomization to gather feasibility and acceptability data for this design and to provide a preliminary estimate of the effect size against a comparator, to inform the planning of a larger trial. Consideration should be given to appropriate control conditions for piloting, for example wait‐list control, or alternative treatment groups, and the implications of these for participant retention requires evaluation. Specific learning points from the current study that inform the next stage of evaluation include to use additional recruitment strategies to access younger age range and non‐students; conduct further pilot work and consultation with individuals with ASD; consider offering online delivery to increase accessibility and consider alternative measures of self‐criticism and self‐compassion. At formal evaluation phase, a larger randomized‐controlled trial and blinded researchers is warranted, to investigate whether changes in self‐esteem and other measures are associated with the intervention, rather than passage of time, expectation of the intervention, placebo effect, or non‐specific therapy factors. Effectiveness could be compared for people with different stigmatized characteristics. A future study could examine possible treatment mediators. Longer follow‐up periods would enable investigation of longer‐term effects of the intervention. The current study used a general population sample so the intervention could be delivered outside NHS settings, a future study could use a clinical sample to examine feasibility and acceptability within NHS context.

### Conclusions

The intervention was found to be feasible and acceptable, and there were large effect sizes for improvements in self‐esteem, which were maintained at follow‐up. These findings indicate the need for further evaluation using a randomized‐controlled design.

## Conflicts of interest

All authors declare no conflict of interest.

## Author contribution

**Katie Langford:** Conceptualization (equal); Data curation (equal); Formal analysis (equal); Investigation (equal); Methodology (equal); Project administration (equal); Resources (equal); Visualization (equal); Writing – original draft (equal); Writing – review & editing (equal). **Katrina McMullen:** Investigation (equal); Methodology (equal); Project administration (equal); Resources (equal); Writing – review & editing (equal). **Livia Bridge:** Methodology (equal); Resources (equal); Writing – review & editing (equal). **Lovedeep Rai:** Methodology (equal); Resources (equal); Writing – review & editing (equal). **Patrick Smith:** Conceptualization (equal); Methodology (equal); Resources (equal); Supervision (equal); Writing – review & editing (equal). **Katharine A. Rimes:** Conceptualization (equal); Methodology (equal); Resources (equal); Supervision (equal); Writing – review & editing (equal).

## Supporting information


**Table S1**. Spearman correlations between pre‐ to post‐treatment changes on clinical and process measures.
**Table S2**. Spearman correlations between pre‐ to post‐treatment changes on Rosenberg Self‐Esteem Scale and each subscale of Discrimination and Prejudice Responses questionnaire.

## Data Availability

Data unavailable due to ethical restrictions.
