# Supplementary material for: A cognitive behavioural intervention for low self‐esteem in young people who have experienced stigma, prejudice, or discrimination: An uncontrolled acceptability and feasibility study
Source: Psychol Psychother. 2021 Aug 29;95(1):34–56. doi: 10.1111/papt.12361 (PMC9292341; doi:10.1111/papt.12361)
Supplement: Supplementary file 1 — Table S1. Spearman correlations between pre‐ to post‐treatment changes on clinical and process measures. Table S2. Spearman correlations between pre‐ to post‐treatment changes on Rosenberg Self‐Esteem Scale and each subscale of Discrimination and Prejudice Responses questionnaire. [file PAPT-95-34-s001.docx]

Table S1. Spearman correlations between pre- to post-treatment changes on clinical and process measures.

|  | **RSES** | **WASAS** | **PHQ-9** | **GAD-7** | **FSCRS-IS** | **FSCRS-HS** | **FSCRS-RS** | **SCS** |
| --- | --- | --- | --- | --- | --- | --- | --- | --- |
| **RSES** |  |  |  |  |  |  |  |  |
| **WASAS** | -.60** |  |  |  |  |  |  |  |
| **PHQ-9** | -.46 | .27 |  |  |  |  |  |  |
| **GAD-7** | -.26 | .09 | .30 |  |  |  |  |  |
| **FSCRS-IS** | -.81** | .76** | .19 | .19 |  |  |  |  |
| **FSCRS-HS** | -.64** | .61** | .33 | .29 | .64** |  |  |  |
| **FSCRS-RS** | .66** | -.62** | -.24 | -.06 | -.76** | -.63** |  |  |
| **SCS** | .67** | -.61 | -.12 | -.14 | -.84** | -.57* | .85** |  |

*Spearman’s rho correlations (n=18); *p<.05; **p<.01;* RSES = Rosenberg Self-Esteem Scale; WSAS = Work and Social Adjustment Scale; PHQ-9 = Patient Health Questionnaire; GAD-7 = Generalised Anxiety Disorder; FSCRS-IS = Forms of Self-Criticising/Attacking & Self-Reassuring Scale – Inadequate Self subscale; FSCRS-HS = Forms of Self-Criticising/Attacking & Self-Reassuring Scale – Hated Self subscale; FSCRS-RS = Forms of Self-Criticising/Attacking & Self-Reassuring Scale – Reassured Self subscale; SCS = Self-Compassion Scale

Table S2. Spearman correlations between pre- to post-treatment changes on Rosenberg Self-Esteem Scale and each subscale of Discrimination and Prejudice Responses questionnaire.

|  | **RSES** | **Preparation** | **Raise awareness** | **Avoidance** | **Enjoyable activity** | **Group attachment** | **Secrecy** | **Self-Reliance** | **Distancing** | **Rumination** | **Resignation** | **Blame** |
| --- | --- | --- | --- | --- | --- | --- | --- | --- | --- | --- | --- | --- |
| **RSES** |  |  |  |  |  |  |  |  |  |  |  |  |
| **Preparation** | .20 |  |  |  |  |  |  |  |  |  |  |  |
| **Raise awareness** | .21 | .41 |  |  |  |  |  |  |  |  |  |  |
| **Avoidance** | .05 | .35 | .21 |  |  |  |  |  |  |  |  |  |
| **Enjoyable activity** | .46 | -.18 | .62** | -.07 |  |  |  |  |  |  |  |  |
| **Group attachment** | .12 | .12 | -.17 | .57* | -.15 |  |  |  |  |  |  |  |
| **Secrecy** | .00 | -.05 | -.49 | .31 | -.16 | .34 |  |  |  |  |  |  |
| **Self-Reliance** | .07 | .12 | .21 | .41 | -.08 | .24 | .05 |  |  |  |  |  |
| **Distancing** | -.23 | .03 | -.53 | .22 | -.43 | .29 | .15 | -.14 |  |  |  |  |
| **Rumination** | -.51* | .05 | -.25 | .12 | -.56* | -.13 | .27 | .02 | -.12 |  |  |  |
| **Resignation** | -.24 | -.10 | -.33 | -.07 | -.36 | -.07 | .16 | .24 | .14 | .25 |  |  |
| **Blame** | .58* | .15 | .19 | -.12 | .41 | 0.8 | -.18 | -.18 | -.15 | -.60** | -.23 |  |

*Spearman’s rho correlations (n=18); *p<.05; **p<.01*
